# Assessment of Clinical Empathy Among Medical Students Using the Jefferson Scale of Empathy-Student Version

**DOI:** 10.7759/cureus.4160

**Published:** 2019-02-28

**Authors:** Shahid H Mirani, Noor A Shaikh, Amber Tahir

**Affiliations:** 1 Surgery, Ghulam Mohammad Mahar Medical College and Hospital, Sukkur, PAK; 2 Pediatric Surgery, Ghulam Mohammad Mahar Medical College and Hospital, Sukkur, PAK; 3 Internal Medicine, Dow University of Health Sciences, Karachi, PAK

**Keywords:** empathy for clinical perspective, lack of empathy, medical students, psychometric evaluation, medical education, self-assessment, attitude, jefferson scale of empathy

## Abstract

Introduction

Clinical empathy is the ability to comprehend the perspectives, feelings, and situation of the patients. Clinical empathy instills a sense of satisfaction in the patient. It also facilitates the healthcare provider (HCPs) in taking more sincere and logical clinical decisions. Although there have been numerous studied conducted to explore the pattern of clinical empathy among medical students, the results are mixed and not consistent.

Methods

This is a cross-sectional, observational study conducted among medical students of Ghulam Muhammad Mahar Medical College in August 2018. Two hundred and seven out of 500 students of all five years completed the study after informed consent. All students completed the 20-item Jefferson Scale of Empathy-Student Version (JSE-S). Data were entered and analyzed using SPSS version 22 (SPSS Inc, Chicago, IL, USA). The internal consistency of JSE-S was 0.71. Frequencies and percentages were calculated for students’ ages and genders. Mean and standard deviation (SD) were calculated for continuous variables. Group comparisons of the empathy scores were conducted using t-test and one-way analysis of variance (ANOVA). p<0.05 was considered as the significant level.

Results

There were 93 (44.9%) male and 114 (55.1%) female students. Their mean ± SD age was 20.85 ± 2.27 years (range: 17 to 26 years). The mean ± SD empathy score of all students was 98.11 ± 12.31 (range: 20-140). The mean empathy score was categorized according to gender, year of education, and career preference. Females showed a significantly higher empathy score. The lowest empathy was seen for the final year and the highest for the first year. On all three subscales of (JSE-S) - perspective taking, compassionate care, and walking in patients’ shoes - students with “people-oriented” career preference scored higher.

Conclusion

JSE-S is a self-administered and self-perceived inventory, which reports declining empathy in medical students with ascending years of education. Qualitative studies that can assess the empathy levels from the patients’ perspective are the need of the hour to decide whether or not empathy is a real phenomenon.

## Introduction

Clinical empathy has been defined by Mercer and Reynolds as the ability to comprehend the perspectives, feelings, and situation of the patients. Clinically empathic healthcare providers (HCPs) communicate with patients at the level of their understanding in such a manner that is therapeutically helpful to them [1].

Clinical empathy instills a sense of satisfaction in the patient. It also facilitates the HCPs in taking more sincere and logical clinical decisions. These factors indirectly contribute to increased compliance and, eventually, better outcomes [2-3].

Over time, there have been various inventories developed for the assessment of clinical empathy among all levels of medical practitioners. The Jefferson Scale of Empathy (JSE) has gained widespread popularity and interest, particularly for use among medical students. It has a customized version for the students known as the Jefferson Scale of Empathy-Student Version (JSE-S). JSE-S was solely designed to monitor clinical empathy in medical students [4].

Although there have been numerous studies conducted to explore the pattern of clinical empathy among medical students, the results are mixed and not consistent. Some researchers have reported a gradual decline in the graph of empathy [5-6], some showed no change over time [7], and some even reported an increase in overall empathy. Other factors, such as gender difference and choice of specialty, have also been studied in relation to empathy trends [8].

The aim of this study is to measure clinical empathy among medical students of Sukkur, Pakistan, using JSE-S.

## Materials and methods

This is a cross-sectional, observational study conducted among medical students of Ghulam Muhammad Mahar Medical College in August 2018. There were 500 students enrolled in this college at that time (100 students per batch of MBBS). All of them were invited to participate. Informed consent was taken.

A self-administered questionnaire in English was handed over to them. It included two sections. The first section included age, gender, and preferred career choice. Career choices were classified into people-oriented, technology-oriented, and undecided [9]. The second section was the Jefferson Scale of Empathy-Student version. This inventory consists of 20 items, which are responded on a seven-point Likert scale with 1 being strongly disagree and 7 being strongly agree for positively responded items and 1 being strongly agree and 7 being strongly disagree otherwise. Therefore, the scores ranged from 20 till 140. Higher scores indicate greater empathy. It has been categorized into three subscales, including perspective taking (10 positively responded items), compassionate care (eight negatively responded items), and walking in patient’s shoes (two negatively responded items) [9]. Permission to use the questionnaire was obtained [4]. It has been previously validated [10].

Statistical analysis was performed in SPSS Version 22 (SPSS Inc, Chicago, IL, USA). The internal consistency of JSE-S was 0.71. Frequencies and percentages were calculated for students’ ages and genders. Mean and standard deviation (SD) were calculated for continuous variables. Group comparisons of the empathy scores were conducted using t-test and one-way analysis of variance (ANOVA). p<0.05 was considered as the significant level.

## Results

The study was completed by 207 medical students (participation ratio: 41.4%). There were 93 (44.9%) male and 114 (55.1%) female students. There were 50 (24.15%) first-year students, 42 (20.2%) second year, 48 (23.18%) third year, 37 (17.87%) fourth year, and 30 (14.4%) fifth year. Their mean ± SD age was 20.85 ± 2.27 years (range: 17 to 26 years). The mean ± SD empathy score of all students was 98.11 ± 12.31 (range: 20-140). The mean empathy score was categorized according to gender, year of education, and career preference. Females showed a significantly higher empathy score. The lowest empathy was seen for the final year and the highest for the first year (Table 1).

**Table 1:** Mean empathy score of the students categorized according to their genders and year of education (n=207)

Gender	Frequency (%)	Empathy Score (mean ± SD)	P-Value
Male	93 (44.9%)	96.38 ± 14.45	0.004*
Female	114 (55.1%)	102.21 ± 13.30	
Year of education			
First	50 (24.15%)	103.21 ± 13.30	0.001**
Second	42 (20.2%)	100.45 ± 11.50	
Third	48 (23.18%)	101.40 ± 12.90	
Fourth	37 (17.87%)	97.18 ± 11.18	
Fifth	30 (14.4%)	92.76 ± 14.26	
Career preference			
People-oriented	104 (50.2%)	104.21 ± 11.41	0.00**
Tech-oriented	49 (23.6%)	93.17 ± 14.27	
Undecided	54 (26.1%)	97.21 ± 12.67	

**Figure 1:**
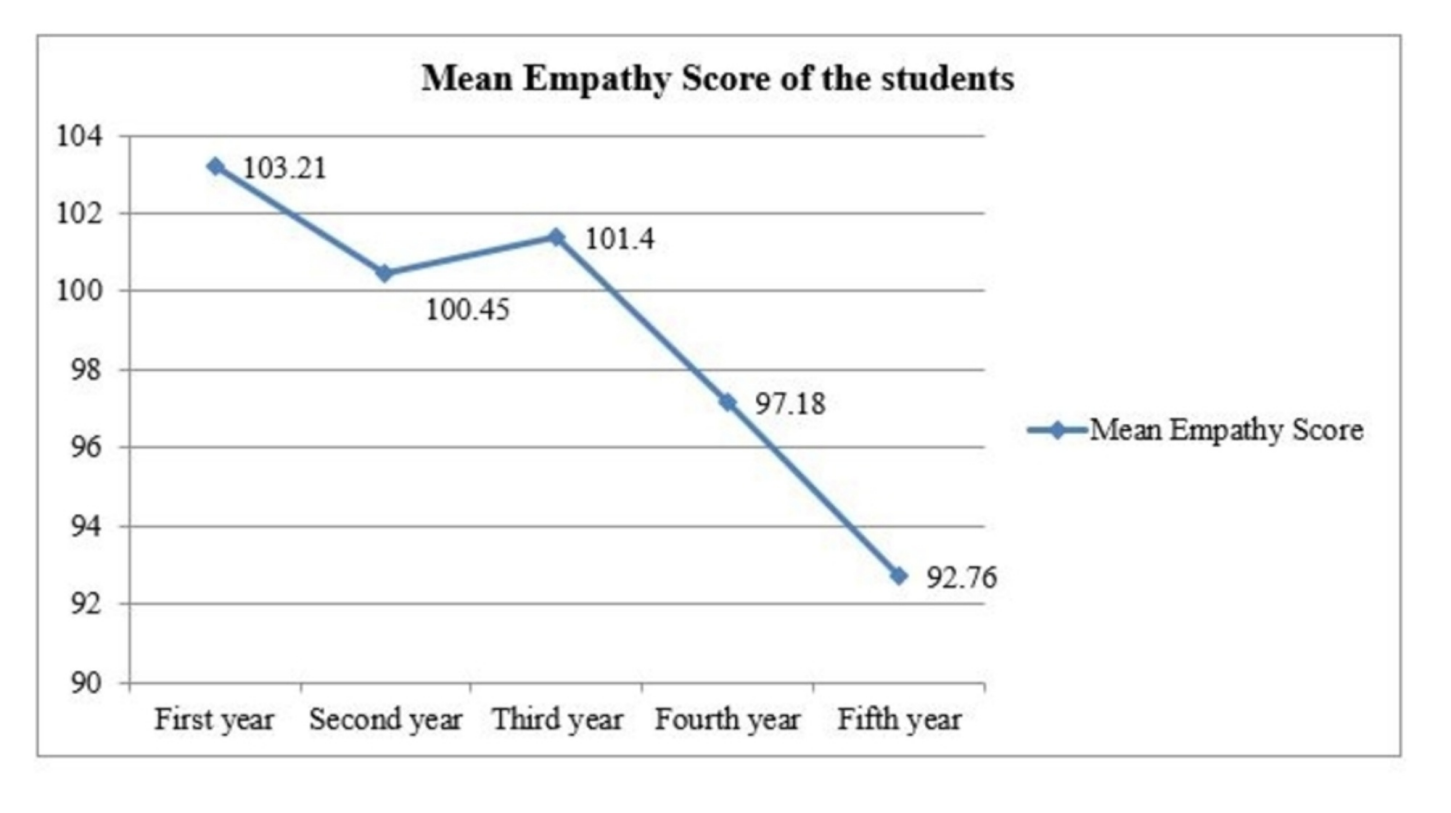
Line graph showing the mean empathy score according to the year of education

The pattern of declining empathy scores over the years of education with a slight rise in the third year is shown in Figure 1.

The JSE-S was subcategorized into three domains - perspective taking, compassionate care, and walking in the shoes of the patients. The mean empathy scores on these three domains were categorized according to the gender, years of education, and the future career preference of the students. The results are shown in Table 2. Perspective taking was higher in women, third-year students, and students who were indecisive about their career choice. Compassionate care scores were higher in women, first-year students, and students with people-oriented career choices. The mean score of walking in patient’s shoes was higher in women, fifth-year students, and students with people-oriented career choices (Table 2).

**Table 2:** Mean empathy score on each subscale categorized according to the genders and year of education of the students (n=207)

	Perspective taking	Compassionate care	Walking in patient’s shoes
Gender			
Male	49.36 ± 5.05	38.74 ± 5.71	7.24 ± 3.01
Female	58.25 ± 11.25	39.48 ± 4.07	8.48 ± 2.89
Year of education			
First	51.87 ± 9.05	39.63 ± 5.82	7.16 ± 3.46
Second	50.85 ± 10.74	38.54 ± 6.94	7.38 ± 2.98
Third	53.98 ± 7.41	39.52 ± 5.55	7.25 ± 2.89
Fourth	49.02 ± 9.40	39.25 ± 7.02	7.10 ± 2.74
Fifth	48.75 ± 6.84	37.07 ± 4.96	7.37 ± 3.88
Career preference			
People-oriented	49.05 ± 7.01	40.28 ± 5.87	8.01 ± 2.11
Technology-oriented	46.66 ± 6.04	39.88 ± 5.97	7.95 ± 2.84
Undecided	48.75 ± 6.84	36.58 ± 7.11	7.69 ± 2.96

## Discussion

This study reports moderate clinical empathy among medical students of all academic years. There was a gradual decline in clinical empathy with ascending years of education. Females were more empathic than males.

The score of overall empathy reported in this study was 98.11 ± 12.31. It was comparable to a study published in India in 2017, in which the mean empathy score calculated via JSE-Student version was 96.01 [8]. Empathy among medical students in Karachi, Pakistan, has been reported to be 42 ± 9.60 for females and 38.7 ± 9.35 for males using the Empathy Quotient Questionnaire [7].

In this study, the mean empathy score was highest in first-year students and lowest in final-year students. This finding is consistent with Chattarjee et al. [8]. Shashikumar et al. [5] and Mostafa et al. [9] have also endorsed a gradual decline in empathy with increasing age or year of education. Studies have attributed many factors to this consistent finding. The stress of academic performance, long work hours [11], lack of quality sleep, and increased responsibilities with age [12] are some factors that contribute to declining empathy among older individuals. However, a Pakistani study conducted by Bangash AS et al. [7] failed to find any association between empathy and age or medical year. The different findings regarding the correlation of year of education and empathy can be attributed to different teaching set-ups in different countries and communities. The mean score on each subscale in this study was also comparable to regional studies [8].

This study also reported higher overall empathy among female students than male students. This result was consistent with both Chatterjee A et al. [8] and Bangash AS et al. [7]. This difference predilection has been explained by Christov-Moore et al. [13]. Based on the neurobiological foundations of empathy, it has been revealed that there are gender differences in the basic networking of cognitive and affective empathy. It also says that the difference may be due to qualitative variance in the manner of integrating emotional information between these two genders and assimilating it into the decision-making process [13].

Now that declining empathy is a well-established outcome, the focus is to be shifted one step ahead. Suitable educational and vocational programmes must be modulated to enhance empathy. Professors and postgraduates must act as empathic role models for the young generation to instill empathic skills, foster teamwork, and lead them to be ideal professionals. In the future, studies that can successfully comprehend the reasons governing gender difference in empathy levels are required. JSE-S is a self-administered and self-perceived inventory. Studies that can assess the empathy levels from the patients’ perspective are the need of the hour, to decide whether or not empathy is a real phenomenon.

## Conclusions

Medicine is a field where empathy is of grave importance, as this field, at its core, is there to serve humanity and those who are suffering. It is thus very important that we pay attention to nurture our medical students to have empathy rather than lose it under the stress of academic performance. The decreasing empathy level with age and year is concerning and studies to find the cause of this shall be conducted. Various workshops shall be conducted to maintain empathy levels among students. Following students annually, from their first year to graduation, to find the true representation of empathy score should also be considered.
